# The risk of ventricular arrhythmias in a Dutch CRT population: CRT-defibrillator versus CRT-pacemaker

**DOI:** 10.1007/s12471-015-0800-8

**Published:** 2016-01-21

**Authors:** I. A. H. ter Horst, J. van ’t Sant, S. C. Wijers, M. A. Vos, M. J. Cramer, M. Meine

**Affiliations:** Department of Cardiology, University Medical Center Utrecht, Heidelberglaan 100, 3508 GA PO Box 85500, Utrecht, The Netherlands; Department of Cardiology, St. Antonius Hospital Nieuwegein, Nieuwegein, The Netherlands; Department of Medical Physiology, University Medical Center Utrecht, Utrecht, The Netherlands

**Keywords:** Cardiac resynchronisation therapy, Ventricular arrhythmias, Appropriate ICD

## Abstract

**Background:**

Patients eligible for cardiac resynchronisation therapy (CRT) have an indication for primary prophylactic implantable cardioverter defibrillator (ICD) therapy. However, response to CRT might influence processes involved in arrhythmogenesis and therefore change the necessity of ICD therapy in certain patients.

**Method:**

In 202 CRT-defibrillator patients, the association between baseline variables, 6-month echocardiographic outcome (volume response: left ventricular end-systolic volume decrease < ≥15 % and left ventricular ejection fraction (LVEF) ≤ >35 %) and the risk of first appropriate ICD therapy was analysed retrospectively.

**Results:**

Fifty (25 %) patients received appropriate ICD therapy during a median follow-up of 37 (23–52) months. At baseline ischaemic cardiomyopathy (hazard ratio (HR) 2.0, *p* = 0.019) and a B-type natriuretic peptide level > 163 pmol/l (HR 3.8, *p* < 0.001) were significantly associated with the risk of appropriate ICD therapy. After 6 months, 105 (52 %) patients showed volume response and 51 (25 %) reached an LVEF > 35 %. Three (6 %) patients with an LVEF > 35 % received appropriate ICD therapy following echocardiography at ± 6 months compared with 43 patients (29 %) with an LVEF ≤ 35 % (*p* = 0.001). LVEF post-CRT was more strongly associated to the risk of ventricular arrhythmias than volume response (LVEF > 35 %, HR 0.23, *p* = 0.020).

**Conclusion:**

Assessing the necessity of an ICD in patients eligible for CRT remains a challenge. Six months post-CRT an LVEF > 35 % identified patients at low risk of ventricular arrhythmias. LVEF might be used at the time of generator replacement to identify patients suitable for downgrading to a CRT-pacemaker.

## Introduction

The majority of patients eligible for cardiac resynchronisation therapy (CRT) also have an indication for primary prophylactic implantable cardioverter defibrillator (ICD) therapy based on their depressed left ventricular ejection fraction (LVEF ≤ 35 %) [1]. Although LVEF is the strongest predictor of ventricular arrhythmias in patients without a history of ventricular arrhythmias, its value in patients eligible for CRT is questionable. CRT aims to improve LVEF and induces reverse remodelling of the left ventricle. Both reverse remodelling and overcrossing a certain threshold in LVEF post-CRT have previously been linked to a decreased risk of ventricular arrhythmias, but inconclusively [2–11]. As a CRT-defibrillator (CRT-D) device is much more expensive than a CRT-pacemaker (CRT-P) and the additional ICD induces the risk of inappropriate ICD therapy, it is clinically relevant to identify those patients at low risk of ventricular arrhythmias prior to CRT-D implant or at the time of CRT-D generator replacement [12].

Therefore, in the current study we aimed to (I) identify baseline predictors of appropriate ICD therapy after the start of CRT; (II) assess whether we can confirm the link between LVEF improvement and reverse remodelling and a lower risk of ventricular arrhythmias; and (III) which echocardiographic outcome parameter, volume response or absolute LVEF, is the best predictor of ventricular arrhythmias and therefore should be used for risk stratification at time of replacement.

## Method

### Study design and population

This retrospective study analysed data from all patients who received either a de novo CRT-D or a CRT-D upgrade at the University Medical Center of Utrecht (UMCU) in the period 2005–2011. Only patients receiving an ICD for primary prevention were included in the analysis, thereby excluding upgrade patients with prior appropriate ICD therapy. Indication for CRT was based on European Society of Cardiology (ESC) guidelines, [13] evidence-based medicine and inclusion criteria of CRT studies [14]. Patients were included if echocardiographic data were available and analysable 1 month before and 6 months after CRT implantation.

### Clinical, laboratory and electrocardiography data

Baseline clinical and laboratory data were collected by reviewing hospital records.

Electrocardiographic characteristics as bundle branch block and QRS duration were analysed on pre-implantation electrocardiograms (ECG) using criteria set by the American Heart Association to define bundle branch block morphology [15].

### Echocardiography data

Echocardiographic studies were performed using a Vivid 7 (General Electric, Milwaukee, USA). Patients were imaged in the left lateral decubitus position. At baseline, volumes, LVEF, and interventricular mechanical delay (IVMD) were assessed. Six months after device implantation, volumes and LVEF were evaluated again during biventricular pacing. LV volumes and LVEF were measured according to Simpson’s biplane method. Measurements were performed three times and averaged. IVMD was defined as the timing difference between opening of the aortic valve and the pulmonary valve. Doppler flows over the pulmonary and aortic valve were recorded and time from Q to onset of flow was assessed for both valves.

### Echocardiographic outcome

Echocardiographic outcome groups were based on a relative decrease in LV end-systolic volume of < ≥15 % (non-responders vs. volume responders respectively), and overcrossing the threshold in absolute LVEF for primary prophylactic ICD therapy of 35 % after 6 months of CRT [16].

### Endpoint

The endpoint was the occurrence of first appropriate ICD therapy during follow-up, including both anti-tachycardia pacing and shock, with notification of time until first appropriate ICD discharge. Episodes of ICD therapy were analysed by experienced cardiologists. Follow-up started directly after implant to assess baseline predictors of appropriate ICD therapy. Ventricular arrhythmias episodes occurring before the 6-month echocardiography were excluded to assess the best echocardiographic outcome predictor of ICD therapy. Patient-specific follow-up ended with the last ICD check-up and the end of all follow-up was the end of December 2013. ICD device settings were collected from the electronic patient files.

### Statistical analysis

IBM SPSS statistics 20.0 (Chicago, Illinois) was used for statistical analysis. Continuous variables were tested for normal distribution by Kolmogorov-Smirnov tests. Normally distributed variables are presented as mean with standard deviation and compared using Student’s T-test. In case of skewed distribution, variables are presented as median with interquartile range (IQR) and compared using the Mann-Whitney U test. Categorical variables are presented as frequencies with percentages and compared using Pearson’s chi-square test.

To assess baseline predictors of appropriate ICD therapy univariate Cox regression analysis was performed. LVEF at baseline, QRS duration and IVMD were dichotomised based on cut-offs that were previously used in the literature: 20 %, 150 and 40 ms, respectively [17]. Other continuous variables were dichotomised by using the optimal cut-off in the receiver operating characteristic (ROC) curves of their association with first appropriate ICD therapy. Correlation between variables was tested using Pearson’s correlation. In case of a significant correlation coefficient > 0.4 the variable with the strongest relation to appropriate ICD therapy was used for the multivariate model. Variables showing a *p* < 0.2 were placed in a multivariate Cox regression model. Echocardiographic outcome parameters were associated with the risk of (first) appropriate ICD therapy using only those events which occurred during follow-up starting ± 6 months after echocardiography was performed. Kaplan-Meier hazard functions were used to determine the association between volume response and reaching an LVEF > 35 % and first appropriate ICD therapy, and significance was assessed by log rank. Subsequently, a multivariate Cox regression model was set up to assess independent association of echocardiographic outcome parameters and the occurrence of appropriate ICD therapy. Both multivariate Cox regression models were performed using backward stepwise regression. Baseline characteristics associated with the outcome measurement predictive of the lowest risk of appropriate ICD therapy were evaluated by performing a backward logistic regression analysis.

## Results

### Study population and baseline characteristics

Detailed baseline characteristics are listed in Table 1. The final study population consisted of 202 patients. Median age was 67 [14] years, 64 % (*n* = 129) male gender, mean LVEF 21 ± 6 %, and 45 % (*n* = 91) ischaemic cardiomyopathy. Mean QRS duration was 165 ± 22 ms, 59 % (*n* = 120) typical left bundle branch block and 11 % (*n* = 22) paced from the right ventricle.

**Table 1:** Baseline characteristic comparison based on echocardiographic outcome groups assessed after six months of CRT

Variables	Volume non-responders (*N* = 97)	Volume responders (*N* = 105)	*P*-value	LVEF ≤ 35 % (*N* = 151)	LVEF > 35 % (*N* = 51)	*P*-value
Age, (median, IQR) yrs	67 (69–74)	67 (60–73)	0.884	67 (60–73)	65 (57–73)	0.659
Males (%)	67 (69)	62 (59)	0.138	102 (68)	27 (53)	0.060
ICMP (%)	55 (57)	36 (34)	0.001	77 (51)	14 (28)	0.003
NYHA III–IV (%)	69 (81)	69 (76)	0.388	111 (84)	27 (61)	0.002
NYHA I (%)	1 (1)	1 (1)		0 (0)	2 (5)	
NYHA II (%)	15 (18)	21 (23)		21 (16)	15 (34)	
Creatinine (median, IQR) µmol/L	114 (93–152)	110 (83–137)	0.018	114 (91–144)	104 (83–129)	0.048
BNP (median, IQR) pmol/L	137 (71–256)	83 (40–194)	0.004	132 (71–262)	45 (28–99)	< 0.001
Diuretics (%)	88 (91)	93 (89)	0.759	139 (93)	45 (82)	0.034
Beta-blocker (%)	80 (83)	86(83)	0.968	123 (82)	43 (84)	0.707
ACE inhibitors (%)	69 (71)	75 (72)	0.877	108 (72)	36 (71)	0.847
ARB (%)	24 (25)	28 (27)	0.724	35 (25)	15 (29)	0.504
Amiodaron (%)	12 (12)	12 (12)	0.856	19 (13)	5 (10)	0.586
Digoxin (%)	24 (25)	22 (21)	0.545	33 (22)	13 (26)	0.608
Sotalol (%)	4 (4)	5 (5)	0.815	6 (4)	3 (6)	0.574
Echocardiography						
LVEF (mean ± SD) %	20 ± 6	21 ± 6	0.470	20 ± 6	24 ± 6	< 0.001
LVESV (median, IQR) ml	182 (138–230)	182 (136–228)	0.553	194 (152–239)	136 (104–186)	< 0.001
LVEDV (median, IQR) ml	232 (177–278)	224 (174–278)	0.514	243 (196–293)	174 (146–229)	< 0.001
IVMD (mean ± SD) ms (*N* = 189)	36 ± 23	56 ± 27	< 0.001	41 ± 27	60 ± 24	< 0.001
Electrocardiography						
History of AF (%)	36 (37)	29 (28)	0.149	45 (32)	17 (33)	0.838
RV paced (%)	12 (12)	10 (10)	0.516	17 (11)	5 (10)	0.773
QRSd (mean ± SD) ms	161 ± 22	170 ± 21	0.004	166 ± 22	165 ± 22	0.891
LBBB (%)	45 (46)	75 (71)	< 0.001	87 (58)	33 (65)	0.373

### ICD settings

Mean heart rate cut-off for the ventricular fibrillation zone was 231 ± 12 beats per minute (bpm). In 193 patients also a ventricular tachycardia zone in which ICD therapy was delivered was set at a median of 180 ± 9 bpm.

### Primary endpoint

Total follow-up had a median duration of 37 (IQR 18–46) months in which 25 % (*n* = 50) received appropriate ICD therapy. During the initial 6 months after implant, 8 patients received appropriate ICD therapy of whom 4 had another episode of appropriate ICD therapy during later follow-up. Therefore, a total of 46 (23 %) patients received appropriate ICD therapy after echocardiographic outcome assessment at ± 6 months. Thirty-nine (85 %) patients received anti-tachycardia pacing and 7 (15 %) received shock therapy as first appropriate ICD therapy. Eighteen (46 %) patients who received anti-tachycardia pacing as initial appropriate ICD therapy received appropriate shock therapy after unsuccessful pacing or later during follow-up.

### Baseline predictors of first appropriate ICD therapy

Univariable and multivariable hazard ratios (HR) for the occurrence of appropriate ICD therapy for baseline characteristics are listed in Table 2. BNP > 163 pmol/l and ischaemic cardiomyopathy were independently associated with a significantly higher risk of ventricular arrhythmias, HR 3.8 *p* < 0.001 and HR 2.0, *p* = 0.019, respectively. Four (7 %) out of 61 patients with both a BNP ≤ 163 ml at baseline and non-ischaemic cardiomyopathy received appropriate ICD therapy.

**Table 2:** Uni- and multivariate Cox regression analyses; Baseline characteristics and 6 months echocardiographic outcome measurements associated with appropriate ICD therapy

	Univariate		Multivariate	
	HR (95 % CI)	*P*-value	HR (95 % CI)	*P*-value
**Total first events (** *N* **= 50)** FU start after implant				
Male	1.60 (0.87–2.95)	0.129		
ICMP	1.58 (0.90–2.75)	0.109	2.00 (1.21–3.55)	0.019
Creatinine > 91 (µmol/L)	1.75 (0.90–3.44)	0.102		
BNP > 163 (pmol/L)	3.18 (1.81–5.60)	< 0.01	3.77 (2.10–6.79)	< 0.001
LVEDV > 249 ml	1.80 (1.03–3.13)	0.039		
**Total (first) events (** *N* **= 46)** FU start after 6 months echocardiography				
6 month—Volume response (≥ 15 %)	0.172 (0.05–0.55)	0.003	0.57 (0.30–1.08)	0.086
6 month—LVEF > 35 %	0.378 (0.20–0.70)	0.002	0.23 (0.07–0.80)	0.020

*BNP* B-type natriuretic peptide, *CI* confidence interval, *FU* follow up, *HR* hazard ratio, *ICMP* ischemic cardiomyopathy, *LVEDV* left ventricular end diastolic volume, *LVEF* left ventricular ejection fraction.

### Echocardiographic outcome and first appropriate ICD therapy

**Fig. 1:**
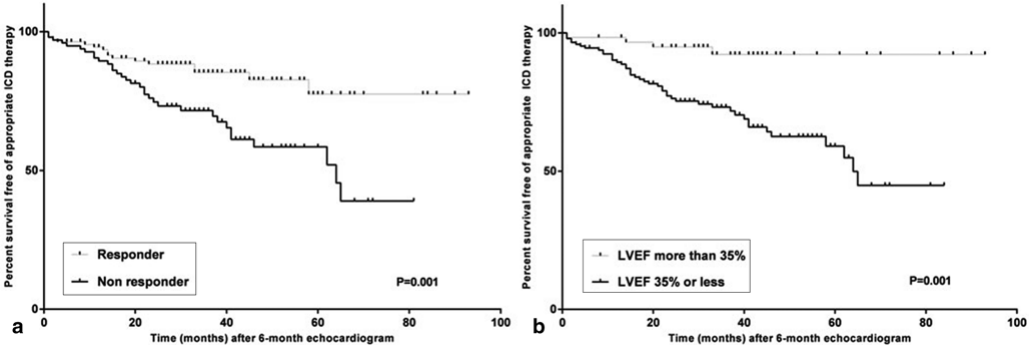
Kaplan-Meier plots of percent survival free of appropriate ICD therapy after 6-month echocardiogram by (**a**) volume response and (**b**) LVEF ≤ > 35 %

After 6 months of CRT, 105 (52 %) patients showed volume response. Fifteen (14 %) responders received appropriate ICD therapy compared with 31 (32 %) non-responders (*p* = 0.001) (Fig. 1a). Fifty-one (25 %) patients reached an LVEF > 35 % after 6 months of which three (6 %) received appropriate ICD therapy compared with 43 patients (29 %) with an LVEF ≤ 35 % (*p* = 0.001, Fig. 1b*)*. Four (4 %) patients who were defined as volume responders at 6 months received appropriate ICD therapy prior to this echocardiogram. None of the patients with an LVEF > 35 % after 6 months received appropriate ICD therapy during the initial 6 months.

Of the volume responders, 44 % reached an LVEF > 35 % while 90 % of patients with an LVEF > 35 % after 6 months were also defined as volume responders (≥ 15 %). Multivariate analysis of 6-month echocardiographic outcome variables showed only LVEF > 35 % to be significantly associated with a lower risk of ventricular arrhythmias (HR 0.23, 95 % CI 0.07–0.78, *p* = 0.020) (Table 2).

Factors independently associated with an LVEF > 35 % after 6 months of CRT were non-ischaemic cardiomyopathy (OR 2.6 95 % CI 1.12–5.97, *p* = 0.026), a higher baseline LVEF (OR 1.16, 95 % CI 1.08–1.24, *P* < 0.001) and a longer IVMD (OR 1.03, 95 % CI 1.01–1.04, *p* < 0.001) (Table 3).

**Table 3:** Multivariate logistic regression model; factors associated with LVEF > 35 % at 6 months

Factors associated with LVEF > 35 % after 6 months of CRT			
	OR	95 % CI	*P*-value
NICMP	2.590	1.123–5.972	0.026
LVEF pre CRT (%)	1.158	1.084–1.237	< 0.001
IVMD pre CRT (ms)	1.025	1.009–1.042	< 0.001

Logistic regression model, corrected for gender and diuretic use.

*BNP* B-type natriuretic peptide, *CI* coincidence interval, *IVMD* interventricular mechanical delay, *LVEF* left ventricular ejection fraction, *NICMP* non-ischemic cardiomyopathy.

## Sub-analysis aetiology of heart failure

Twenty-seven (30 %) patients with ischaemic cardiomyopathy received appropriate ICD therapy compared with 23 (21 %) with non-ischaemic cardiomyopathy (*p* = 0.105). Thirty-six (40 %) patients with ischaemic cardiomyopathy showed volume response and only 14 (15 %) reached an LVEF > 35 %. Eight (22 %) ischaemic volume responders received appropriate ICD therapy compared with 19 (35 %) non-responders (*p* = 0.301). For those ischaemic patients with an LVEF > 35 % after 6 months of CRT, there was a trend towards a lower risk of appropriate ICD therapy (*p* = 0.062).

### Inappropriate ICD therapy and mortality

A total of 21 (10.4 %) patients received inappropriate ICD therapy during follow-up. Ten (5 %) patients received an inappropriate shock including two responders: one of these two patients reached an LVEF > 35 %. During follow-up 39 (19.3 %) patients died: 27 (27.8 %) non-responders, and 12 (11.4 %) responders (*p* = 0.002). No patients with an LVEF > 35 % died during follow-up. Annual mortality rates were 10.2 % in non-responders, 3.4 % in responders, 8.3 % in patients with an LVEF ≤ 35 % and 0 % in patients with an LVEF > 35 % after 6 months of CRT.

## Discussion

In the current study we showed that ischaemic cardiomyopathy and a BNP level > 163 pmol/l at baseline were associated with an increased risk of ventricular arrhythmias after CRT implantation. Other studies evaluating baseline predictors of ventricular arrhythmias in a CRT population found variable results but identified gender, no beta-blocker or ACE inhibitor use, severely decreased LVEF and NYHA class IV as predictors of ventricular arrhythmias [18–20]. Friedman et al. found that LV end-diastolic diameter was a strong predictor of ventricular arrhythmias in a CRT population even when corrected for reverse remodelling after CRT [20]. Moreover, Van der Heijden et al. found LV end-systolic volume > 130 ml predictive of appropriate ICD therapy [21]. Although we could not confirm these results we did see a trend for larger cardiac volumes at baseline to be associated with a higher risk of appropriate ICD therapy. Furthermore, most of these baseline predictors of ventricular arrhythmias after CRT implantation seem to relate to the degree of heart failure prior to implant, as does BNP level, which was a predictor in our population. The variability among studies indicates that prior to implant it is hard to identify valid predictors of ventricular arrhythmias, probably caused by the dynamic nature of CRT which induces changes in baseline variables associated with processes important in arrhythmogenesis, such as wall stress [4], oxygen consumption and supply [22], and neurohormonal activation [23].

### CRT and arrhythmogenesis

Regarding our second aim, we found that patients with a favourable echocardiographic outcome at 6 months had a significantly lower risk of ventricular arrhythmias compared with those showing a less favourable outcome. Although the majority of previous single-centre studies analysing the effect of reverse remodelling after CRT implantation on the risk of ventricular arrhythmias found comparable results, there was some controversy. The observed discrepancy might be due to different definitions of volume response, inclusion of both primary and secondary prophylactic ICD patients and different follow-up durations [2–6, 19]. We showed absolute LVEF at 6 months to be a stronger predictor of ventricular arrhythmias than the occurrence of volume response. An explanation might be that volume response only reflects a relative improvement while absolute LVEF after CRT implantation is more strongly associated with pre- and post-CRT cardiac volumes. Sub-analysis of heart failure aetiology showed that volume response in patients with ischaemic cardiomyopathy was not significantly associated with the risk of ventricular arrhythmias while there was a trend towards a significant correlation with LVEF at 6 months and appropriate ICD therapy. These findings might have multiple explanations. First of all, it is known that patients with ischaemic cardiomyopathy respond less to CRT than those with non-ischaemic cardiomyopathy [24]. Moreover, the mechanism of ventricular arrhythmias in ischaemic cardiomyopathy patients is most often a re-entry tachyarrhythmia involving the scar tissue [25]. It is unlikely that response to CRT is of influence on these ventricular arrhythmias. Finally, response to CRT enables the patient to perform at a higher level of activity; in patients with ischaemic cardiomyopathy this could increase the ischaemic events and therefore trigger ischaemia-induced ventricular arrhythmias.

Although the majority of the evidence shows that favourable echocardiographic outcome after CRT is associated with a lower risk of ventricular arrhythmias, this is compared with patients who show a less favourable response to CRT. It is unclear whether CRT itself is antiarrhythmic as most studies did not have an ICD-only group for comparison. Moreover, there are also some suggestions for a proarrhythmic effect based on small case cohorts and reports [26]. Most of these proarrhythmic events after CRT are linked to pacing in scar tissue but it is also possible that reversal of transmural activation pattern, due to LV epicardial pacing, increases repolarisation dispersion and thereby increases the risk of ventricular arrhythmias. Although we did not have an ICD-only group either, previous occurrence of first appropriate ICD therapy among primary prophylactic one- or two-chamber ICD patients implanted in our hospital was analysed by Wijers et al. [27]. Eighteen percent (*N* = 55) (mean LVEF 24 ± 6 %) received appropriate ICD therapy during a follow-up of 31 ± 17 months. As 25 % of our CRT-D patients received appropriate ICD therapy during a median follow-up of 37 months, it seems the overall effect of CRT on arrhythmogenesis is neutral. However, when looking at the occurrence of ventricular arrhythmias in non-responders (32 %) and responders (14 %) it suggests competing proarrhythmic effects in non-responders and antiarrhythmic effects in responders. Although this is in line with the results of the REVERSE study and the MADIT CRT trial, any conclusion based on comparison of these numbers is limited since an adequate baseline comparison was not performed and therefore other patient characteristics could have influenced the results [2, 3].

### CRT-D or CRT-P

The majority of CRT devices implanted in the Netherlands are CRT-D devices [28]. Even though all patients eligible for CRT have an indication for primary prophylactic ICD therapy based on their depressed LVEF, the survival benefit of CRT-D over CRT-P is still matter of debate. In the choice between CRT-D and CRT-P, the 2013 ESC guideline, endorsed by our national society, the NVVC, states that no strict recommendations on device choice can be made due to insufficient evidence from randomised controlled trials. The guideline merely aims to offer guidance regarding the selection of patients for CRT-D versus CRT-P based on clinical condition, device-related complications as inappropriate ICD therapy, and costs [1]. We showed that 10 % of our CRT-D population received inappropriate ICD therapy, which is associated with healthcare costs, and CRT-D device costs are much higher than CRT-P costs. Therefore, careful consideration in the choice of CRT device is warranted. Moreover, basing CRT device choice on pre-CRT clinical condition is questionable as we, and the majority of studies, have shown response to CRT to influence the risk of ventricular arrhythmias.

Only first appropriate ICD therapy was analysed, as we opted that the risk of first appropriate ICD therapy was of most interest in the decision to implant a CRT-D or a CRT-P, because one episode of appropriate therapy can in theory have prevented a sudden cardiac death. Whether the patient receives more appropriate ICD therapy after this episode is of limited additional value in CRT device choice.

Patients with an LVEF > 35 % after CRT implantation were at a low risk (6 %) of appropriate ICD therapy during 37 months of follow-up. Previously, Van Boven et al. [8] actually showed that none of the patients who reached an LVEF ≥ 35 % after 4 months of CRT needed appropriate shock therapy during a follow-up of 3 years. Manfredi et al. [9] and Schaer et al. [10], who used a higher cut-off in LVEF, found similar results and Ruwald et al. [11] showed with 752 patients from the MADIT CRT trial that patients who achieve LVEF normalisation (> 50 %) have a very low absolute and relative risk of ventricular tachyarrhythmias within 2.2 years of follow-up.

Therefore it would be of great value to correctly predict which patients are most likely to reach an LVEF > 35 % as these patients could be candidates for CRT-P implantation. We propose that the characteristics associated with reaching this LVEF in our cohort, namely non-ischaemic cardiomyopathy, higher IVMD, and higher LVEF, could be used as pre-implantation predictors of patients suitable for CRT-P.

Although these characteristics have also been previously linked to response to CRT in other cohorts [29, 30], prediction of echocardiographic outcome has proven to be difficult. As non-responders might actually be at increased risk of ventricular arrhythmias and responders could still be at risk during the initial 6 months the association found might be of more value for the decision to downgrade a CRT-D in case of generator replacement. Meaning that if a patient has an LVEF > 35 % at the time of generator replacement, CRT-D downgrading to a CRT-P could be considered. This consideration should be restricted to patients with non-ischaemic cardiomyopathy as the association between echocardiographic outcome and appropriate ICD therapy was much weaker in ischaemic cardiomyopathy patients. Moreover, it is unclear whether the relationship between LVEF and risk of ventricular arrhythmias at the time of generator replacement, approximately 5 years after implant, is comparable with the relationship found during the currently evaluated period. We recommend further research into this correlation.

## Limitations

A limitation of this retrospective analysis is the preselected population of CRT patients based on the need for paired echocardiography data. For any recommendation on CRT device choice an important limitation is the relatively short follow-up as we do not know what happens to the risk of ventricular arrhythmias after the median 37 months of our follow-up. Some minor limitations are due to the retrospective design and reflect the incomplete data concerning NYHA class and BNP.

## Conclusion

Baseline prediction of the risk of ventricular arrhythmias in patients eligible for CRT remains challenging. After 6 months of CRT, absolute LVEF is the strongest predictor of ventricular arrhythmias. Patients with an LVEF > 35 % at 6 months are at a low risk of appropriate ICD therapy. Therefore LVEF post-CRT could be used to decide on downgrading of a CRT-D to a CRT-P in case of generator replacement.
